# Editorial: Liquid biopsy and new omics technologies in vascular malformations

**DOI:** 10.3389/fgene.2024.1486606

**Published:** 2024-09-10

**Authors:** Massimo Vaghi, Elisa Frullanti, Maria Palmieri

**Affiliations:** ^1^ Radiologia Interventistica, Ospedale Maggiore di Crema, Largo Ugo Dossena, Crema, Italy; ^2^ Chirurgia Vascolare, Ospedale Maggiore di Crema, Crema, Italy; ^3^ Cancer Genomics and Systems Biology Lab, Department of Medical Biotechnologies, University of Siena, Siena, Italy; ^4^ Department of Medical Biotechnologies, Med Biotech Hub and Competence Centre, University of Siena, Siena, Italy

**Keywords:** liquid biopsy, vascular malformations, omics technologies, WES, Cf-DNA, noninvasive techniques, high throughput sequencing

## 1 Introduction

In recent decades there has been a significant growth in the study of vascular anomalies leading to the identification of molecular pathways and genetic mutations responsible for the formation and progression of these pathologies. Vascular malformations (VM) are congenital anomalies of the blood and lymphatic vessels, therefore many times these are already present at birth, while other times appear in childhood and adolescence (Kunimoto et al., 2022). VMs, can appear in any part of the body with wide severity, causing pain, swelling and/or discoloration of the skin, blood clotting problems, organ damage, functional or aesthetic problems. VM are rare and occur in about 1% of all births with a frequency of 1 person in 5,000–10,000 people (Vikkula et al., 2001) for the venous malformations, the most common type of vascular anomalies. To date, the treatments available focus on reducing symptoms through sclerotherapy, catheter embolization, laser treatments and radiation therapy (radiosurgery).

Given the rarity of these vascular anomalies and the number of different types of malformations, diagnoses are often difficult, therefore genomic studies are required. Unfortunately, genetic tests involve painful tissue biopsies which are not always feasible and cause anxiety for the patient. For this reason, liquid biopsy has appeared as a new noninvasive investigation technique, applied to the field of vascular malformations.

Due to the pioneering studies about the application of liquid biopsy for the diagnostic investigation of vascular malformations and the huge resource that Next-generation Sequencing techniques represent, such as the whole exome sequencing (WES) to better investigate the congenital prenatal genetic defects, there is an urgent need to pursue these studies. Here, we report the studies that have been published in this Research Topic to respond to these purposes.

## 2 Pioneering approaches and proven next-generation sequencing to discover genetic defects in different research areas

The case report in this Research Topic presented by Serio et al. contains a interesting insight about a 61 years old female patient, affected by a Klippel Trenaunay Syndrome with a somatic mosaic mutation of *PIK3CA* (p. (E545G)) identified using both cfDNA Next-Generation Sequencing (NGS) liquid biopsy and tissue biopsy. The patient developed a lung bilateral adenocarcinoma arose on *PIK3CA* mutated tissues monitored with cfDNA-NGS liquid biopsy. (Serio et al.).

Mansur et al. present a review on vascular malformations exploring molecular biology pathways for the use of drugs widely studied for the treatment of oncological pathologies and also useful nowadays for the treatment of vascular pathologies following a molecular diagnosis possible only through the pioneering use of liquid biopsy applied to VMs. (Mansur et al.).

In the context of using liquid biopsy as a new emerging technique of NGS, Liu et al. present an original article in which they study miRNAs extracted from saliva exosomes of lung cancer patients and healthy controls. Their results suggest that miRNAs from salivary exosomes could be used as biomarkers for lung cancer prediction and diagnosis. (Liu et al.).

The use of liquid biopsy is just the latest Frontier in a large area of techniques called next-generation that allow us to sequence large genomes in a short time. Among these, the one that has been most considered in common scientific and diagnostic use is certainly the WES. Due to the increased demand for WES and the decreased cost of NGS, this technique requires a generous understanding of how experimental design can improve data interpretation and thus improve biological outcomes. The data report article by Sun et al. assess the impact of seven popular analysis pipelines to understand the influence of these pipelines on WES results. (Sun et al.).

The use of WES for genetic investigations in the prenatal setting has been introduced as a routine practice in the National Health Service (NHS) in England, receiving favorable opinions from both healthcare personnel, technical and scientific researchers and patients as reported in the original research article by Peter et al.

## 3 Conclusion

These positive results are proof that scientific progress on NGS techniques for genetic investigations in different fields, such as oncology, prenatal and vascular malformations as reported here, are going in the right direction and proceeding at a brisk pace.
